# Differences in ball speed and three-dimensional kinematics between male and female handball players during a standing throw with run-up

**DOI:** 10.1186/s13102-015-0021-x

**Published:** 2015-11-18

**Authors:** Ben Serrien, Ron Clijsen, Jonathan Blondeel, Maggy Goossens, Jean-Pierre Baeyens

**Affiliations:** 1 Department Biomechanics, Vrije Universiteit Brussel, Brussels, Belgium; 2 Thim Van Der Laan University College, Landquart, Switzerland; 3 University of Antwerp, ICT-electronics, Antwerp, Belgium; 4 Department Health Sciences, University of Applied Sciences and Arts of Southern Switzerland, Landquart, Switzerland

## Abstract

**Background:**

The purpose of this paper was to examine differences in ball release speed and throwing kinematics between male and female team-handball players in a standing throw with run-up. Other research has shown that this throwing type produces the highest ball release speeds and comparing groups with differences in ball release speed can suggest where this difference might come from. If throwing technique differs, perhaps gender-specific coordination- and strength-training guidelines are in order.

**Methods:**

Measurements of three-dimensional kinematics were performed with a seven-camera VICON motion capture system and subsequent joint angles and angular velocities calculations were executed in Mathcad. Data-analysis with Statistical Parametric Mapping allowed us to examine the entire time-series of every variable without having to reduce the data to certain scalar values such as minima/maxima extracted from the time-series.

**Results:**

Statistical Parametric Mapping enabled us to detect several differences in the throwing kinematics (12 out of 20 variables had one or more differences somewhere during the motion). The results indicated two distinct strategies in generating and transferring momentum through the kinematic chain. Male team-handball players showed more activity in the transverse plane (pelvis and trunk rotation and shoulder horizontal abduction) whereas female team-handball players showed more activity in the sagital plane (trunk flexion). Also the arm cocking maneuver was quite different.

**Conclusions:**

The observed differences between male and female team handball players in the motions of pelvis, trunk and throwing arm can be important information for coaches to give feedback to athletes. Whether these differences contribute to the observed difference in ball release speed is at the present unclear and more research on the relation with anthropometric profile needs to be done. Kinematic differences might suggest gender-specific training guidelines in team-handball.

## Background

Team-handball is a popular and very dynamic team sport with approximate 800.000 teams spread over 183 countries [1]. Looking at the available literature, it is clear that male players produce higher throwing speeds than female players [2–4]. This is a big advantage to score a goal in team-handball because it decreases the reaction time available to the goal keeper. In female team-handball, the goalkeepers have more time to react to the throw. Much time in training is therefore focused on improving throwing speed. Studies with experienced team-handball players [5, 6] have shown only a very small speed-accuracy trade-off and therefore ball speed is the main performance indicating variable for successful throwing towards goal. Besides differences in ball speed, it is important for coaches to know whether there are gender-related differences in coordination. This can guide the composition of training schedules. Wagner et al. recently reviewed individual and team performance in team-handball [7]. They showed that coordination was one of the determinants of ball release speed. In overarm throwing, the so-called proximal-to-distal sequence of joint motions is an important part of the coordination to generate and transfer momentum to the end-effector, in this case the ball [8–10]. Another important aspect for individual performance is the anthropometric profile. The study of van den Tillaar & Ettema [3] specifically looked at gender differences in team-handball players regarding ball release speed, anthropometric profile and isometric strength. The gender difference in throwing speed reported in their study could be almost completely explained by differences in height and fat-free-mass as an approximation for skeletal muscle mass. In a later study [4], this gender difference was approached from the perspective of 3D kinematics (coordination). They calculated several kinematic and temporal variables but major differences were only found for ball release speed and linear end-point velocities of wrist and hand. Very small and mostly non-significant differences in joint angles and timing of certain events were found, leading them to conclude that differences in throwing velocity are not related to different throwing patterns.

On the other hand, many other studies on the 3D kinematics of team-handball throwing which operate within a deterministic approach [11] were quite able to find differences in selected kinematic or temporal variables between players of different competition levels [10, 12], different ages [13] and different throwing-like sports [14]. Keeping in mind that these kind of cross-sectional studies (comparing different groups) cannot be used to make causal inferences to ball release speed, they have identified several variables that indicate different throwing mechanics. Within subject comparisons on team-handball throwing kinematics revealed significant differences between the dominant- and non-dominant arm [15], throwing with different ball weights [16], different types of team-handball throws [17, 18] and different types of wind-up [19]. These studies were thus able to link differences in input parameters (throwing kinematics) to differences in output parameters (ball release speed) based on contrast and correlation statistics (t-tests, ANOVA’s, regression).

Gender differences in throwing kinematics might be present, but classical statistical techniques might not be sensitive enough to detect them. The classical statistical techniques that are used in the studies mentioned above, allow only the use of scalars. The studies in applied biomechanics on team-handball used scalar points (0D) such as maximal or minimal values extracted from kinematic time series (e.g. maximal shoulder endorotation velocity) or the timing of certain key events (e.g. relative timing of initiation of shoulder endorotation). Statistical Parametric Mapping (SPM) is a statistical technique that was developed in the field of neuro-imaging [20] and an SPM software package specific for one-dimensional time series (as are common in biomechanics) was developed by T. Pataky (SPM-1D ©). SPM-1D applies common statistical techniques (ANOVA, *t*-test, regression) on time series so that no information is lost to scalar extractions. Pataky, Robinson & Vanrenterghem [21] found experimental evidence that scalar extraction can bias the analysis by failing to consider the remaining part of the data-set. Any differences in throwing kinematics are not necessarily located around the minima or maxima of time series. SPM calculates the test statistic of interest (F or t-values, etc.) on every node in the time series, but instead of computing a *p*-value for every node, inferential statistics are based on Random Field Theory [22]. The *p*-values represent the probability that a random Gaussian 1D time series with the same smoothness as the observed data would produce a supra-threshold cluster with an extent as large as the observed cluster [21]. A critical test statistic is calculated based on the a-priori alpha-level and the smoothness of the residuals. If the test-statistic field reaches supra-threshold values, a cluster-width inference is computed (in the present applications of SPM, the inference is only based on cluster width and not on the height above the threshold [21]). This technique makes it possible to use the entire dataset and thus pose non-directed research questions.

In this paper, SPM has been used to answer to the research question: “Is there a gender difference in ball release speed and kinematic variables from the trunk and throwing arm in a team-handball standing throw with run-up?”. We hypothesized that male handball players will exhibit a larger ball release speed. Based on the results of several other successful deterministic studies in handball throwing, we hypothesized that gender differences in throwing kinematics to be present as detected by means of SPM.

## Methods

### Subjects

The subjects that participated in this study were experienced male (*n* = 10) and female (*n* = 10) handball players from two different Swiss handball teams (second league, semi-professional). Anthropometric and training data were gathered from all subjects. Male players had a mean (± SD) age of 25.4 ± 4.0 years, training experience of 11.4 ± 4.7 years, height of 1.82 ± 0.05 m and weight of 86.2 ± 12.5 kg. Female players had a mean (± SD) age of 23.7 ± 2.7 years, training experience of 13.1 ± 4.1 years, height of 1.69 ± 0.06 m and weight of 63.7 ± 4.7 kg. Both genders were matched in age and playing experience, but not for weight and height, as these four parameters are difficult to match at the same time. Both groups had one player who was left-handed. All players signed informed consent forms prior to the measurements after an explanation of the procedures. This study was approved by the Vrije Universiteit Brussel ethics committee in cooperation with the Thim Van Der Laan University College.

### Procedures

Following a general and handball specific warm-up, 43 retro reflective markers were attached to the player’s skin on bony landmarks using adhesive double-sided tape (sacrum, T10, C7, 2 markers on the sternum, bilateral: superior iliac spine, acromion, superior scapular angle, epicondylus lateralis and medialis from the humerus, olecranon, styloid processes from radius and ulna, 2^nd^ and 5^th^ dorsal metacarpal head, epicondylus lateralis and medialis from the femur, lateral and medial malleolus. On the throwing arm, 2 plates of four markers were attached to the upper and lower arm. Three additional markers were fixed non-linearly on the ball to detect the center of the ball, this excludes contributions of ball spin to ball release speed. The subjects performed standing throws with run-up (STWR) with the last foot placement at a 7 m-line towards a handball goal. The players were instructed to perform maximal velocity throws towards a cross (2 arms of 40 cm) in the center of a soft mattress (to absorb the ball speed, the mattress was 2 m by 3 m, the size of a handball goal). Players had to throw until three successful throws were performed (ball hit the cross).

### Data collection and data processing

Three-dimensional kinematic data were captured with a 7-camera VICON MX F20 system at 250 Hz (VICON® Peak, Oxford UK). Three-dimensional marker trajectories were reconstructed and gaps were filled in the VICON Nexus 1.8.2 software and smoothed with a fourth order Butterworth filter (zero lag) at a cut-off frequency of 13 Hz, provided in the Nexus® software. Marker coordinates were exported to a .csv file and imported into a custom-made algorithm in Mathcad 14.0 (Parametric Technology Corporation, MA, USA). The origin of the global reference frame (G) was set at the 7 m-line with the positive Y-axis in the direction of the throw, the positive X-axis to the right and an upward positive Z-axis. Ball speed was calculated with the central difference method based on the midpoint of the three markers on the ball. Ball release was defined as the moment where an increase in ball-hand distance markedly occurred [23] at which point, ball speed (norm of the velocity vector) was extracted for statistical analysis. Local reference frames were defined for the upper-arm (UA), the trunk (TR) and the pelvis (PE). Shoulder joint angles were defined as the Euler angles between the UA and TR reference frames in an order of horizontal ab/adduction, ab/adduction and endo/exorotation. Trunk rotation angles were defined as the Cardan angles between TR reference frame relative to the PE reference frame in an order of flexion/extension, left/right lateroflexion and endo/exorotation. The pelvis orientation relative to the global reference frame was calculated with the Cardan angle sequence of forward/backward tilting, lateral tilting and rotation. To account for different approach angles of the subjects, the angle time series of pelvis rotations were normalized to the point of ball release so all pelvis angles become zero at the point of ball release [24]. The elbow angle was calculated through the standard goniometric cosine formula with the orientations of the upper-arm and the lower-arm. All angle-time series were differentiated with respect to time (central difference method) for obtaining the angular velocity time series after the necessary transformations for the Euler/Cardan angles. All variables (*n* = 20) were calculated within a time-span of 500 ms (125 data frames) before ball release and 200 ms (50 data frames) after ball release. The Euler and Cardan rotation sequences yielded no gimbal locks during this time-span. All kinematic variables for the two left-handed players were transformed so the kinematic time series showed the same pattern as for all right-handed players.

### Statistical procedures

**Fig. 1:**
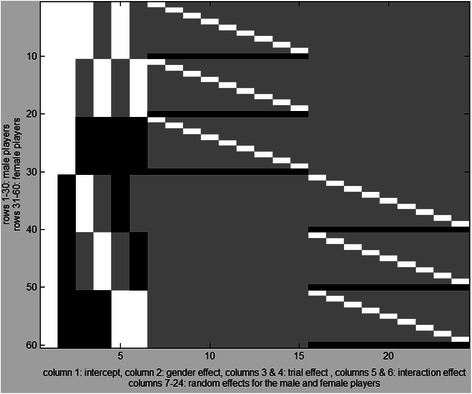
Design matrix for the mixed model ANOVA with effect coding. Colors: white = 1, grey = 0, black = −1. The intercept is represented by column 1. The gender effect is represented by column 2. The within trial effect is represented by columns 3 and 4. Columns 5 and 6 represent the interaction effect. The final columns (7–24) represent the random effects of the i^th^ subject in the j^th^ level of the factor gender

**Fig. 2:**
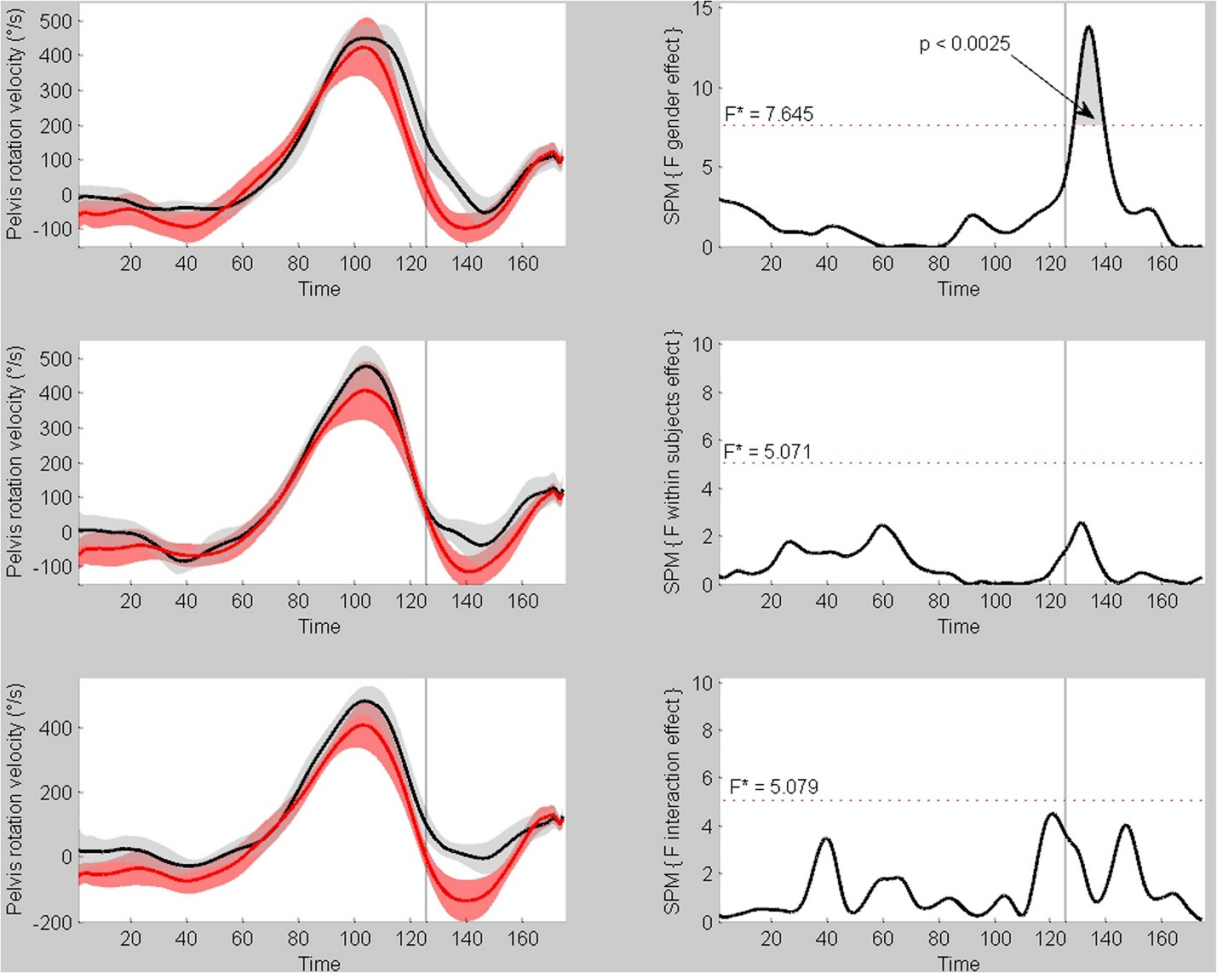
Example of the two-way ANOVA results on the variable Pelvis rotation velocity. Left: mean ± SD for the male (black) and female (red) time series of pelvis rotation velocity throughout the throw (3 trials). The vertical line at time = 125 indicates the point of ball release. Right: results of the three ANOVA’s (gender effect, within subjects effect and interaction effect). The figures contain the critical thresholds (F*) above which a significant effect occurs

All statistical tests were done in Matlab R2013b. At first, we performed a mixed model ANOVA (gender by trial) for ball release speed to test for gender differences, intra-individual variation and interaction effect. Effect sizes (partial η^2^) and power were calculated for every effect. All statistical tests on kinematic variables were performed with the open-source toolbox SPM-1D (© Todd Pataky 2014, version M0.1) that performs Statistical Parametric Mapping on 1-dimensional time-series. We first performed two-way ANOVA SPM{F} tests to see whether there was an interaction effect between gender differences and possible intra-individual variation in the kinematic time-series (3 trials) on every variable. This is a (2×3) mixed model ANOVA. All calculations for the two-way ANOVA’s were executed with a general linear model-approach (GLM) with α = 0.0025 (Bonferroni correction on α = 0.05 for *n* = 20 variables). The design matrix for the full GLM is depicted in Fig. 1. All effects were estimated by comparing the full and reduced GLMs. These two-way ANOVA’s were performed to determine whether a possible gender difference would be dependent on trial-to trial variation. An example of a two-way ANOVA is depicted in Fig. 2. The two-way ANOVA’s for all variables yielded no significant interaction effects and no effects within subjects, therefore we performed two-sample SPM{t} tests (two-sided) on all variables with an a-priori α-level of 0.0025. These t-tests were performed in favor of reporting the SPM{F} field of the between factor (F-test for the gender effect) because of possible non-phasic interactions between the three effects [25]. All *p*-values corresponding to a significant supra-threshold cluster were corrected using a Bonferroni adjustment.

## Results

The results of the scalar mixed model ANOVA on ball release speed is depicted in Table 1. These results indicate a low, non-significant within-subject variability and no interaction effect between gender effect and trial effect. Only the gender effect reached statistical significance and had a high power.

**Table 1:** Ball release speeds and results of the mixed model ANOVA on ball release speed

Means ± SD (m/s)				
	Trial 1	Trial 2		Trial 3
Male players	20.16 ± 2.58	21.05 ± 3.53		20.45 ± 2.72
Female players	15.80 ± 2.63	16.41 ± 1.70		16.33 ± 21.6
Mixed model ANOVA				
	F	*p*-value	Effect size (partial η^2^)	Power (1-β)
Gender effect	15.897	<0.001	0.469	0.965
Trial effect	2.245	0.121	0.111	0.427
Interaction effect	0.282	0.756	0.015	0.091

**Fig. 3:**
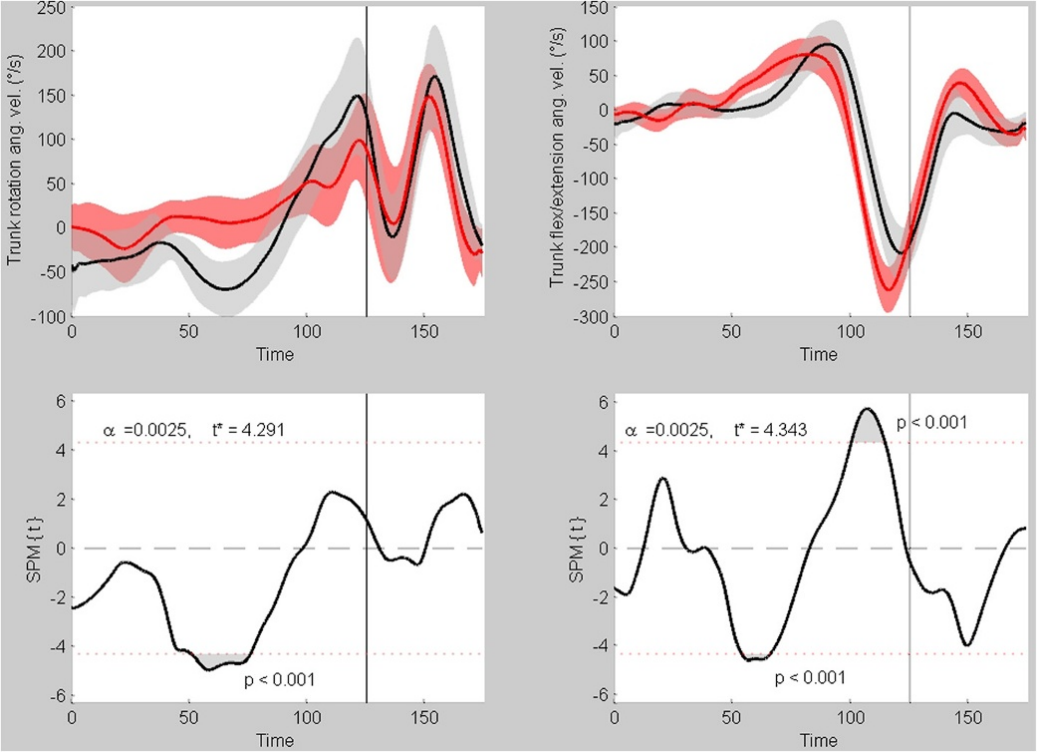
Trunk kinematics. Mean ± SD time series and respective SPM{t} fields for trunk endorotation(+)/exorotation(−) velocity (left) and trunk flexion(−)/extension(+) velocity (right). Black = male, red = female

**Fig. 4:**
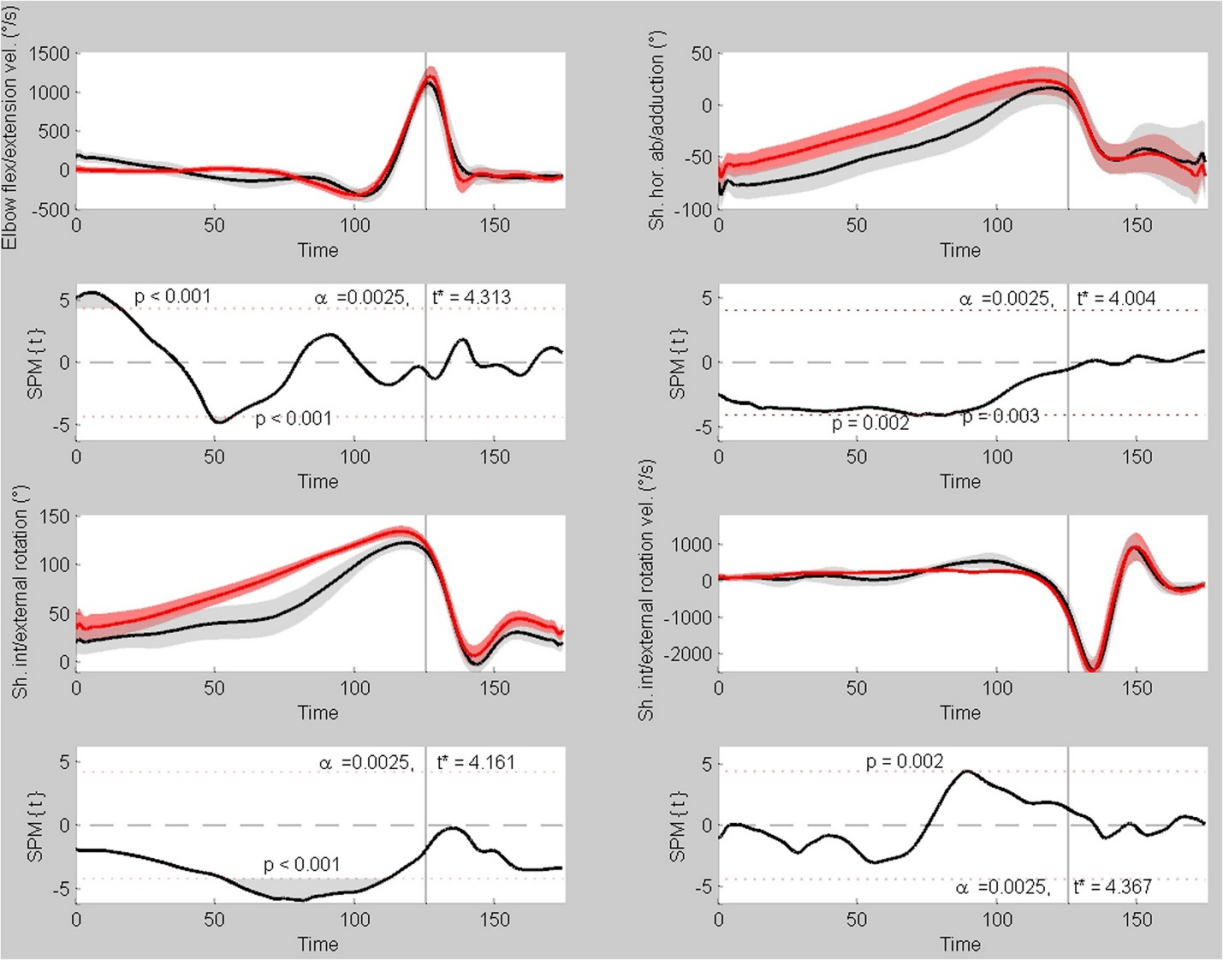
Shoulder and elbow kinematics. Mean ± SD time series with their respective SPM{t} fields below them for elbow flexion(−)/extension(+) velocity (upper left figure), shoulder horizontal ab(−)/adduction(+) (upper right figure), shoulder internal(−)/external(+) rotation (lower left figure) and shoulder internal(−)/external(+) rotation velocity (lower right figure). Black = male, red = female

**Fig. 5:**
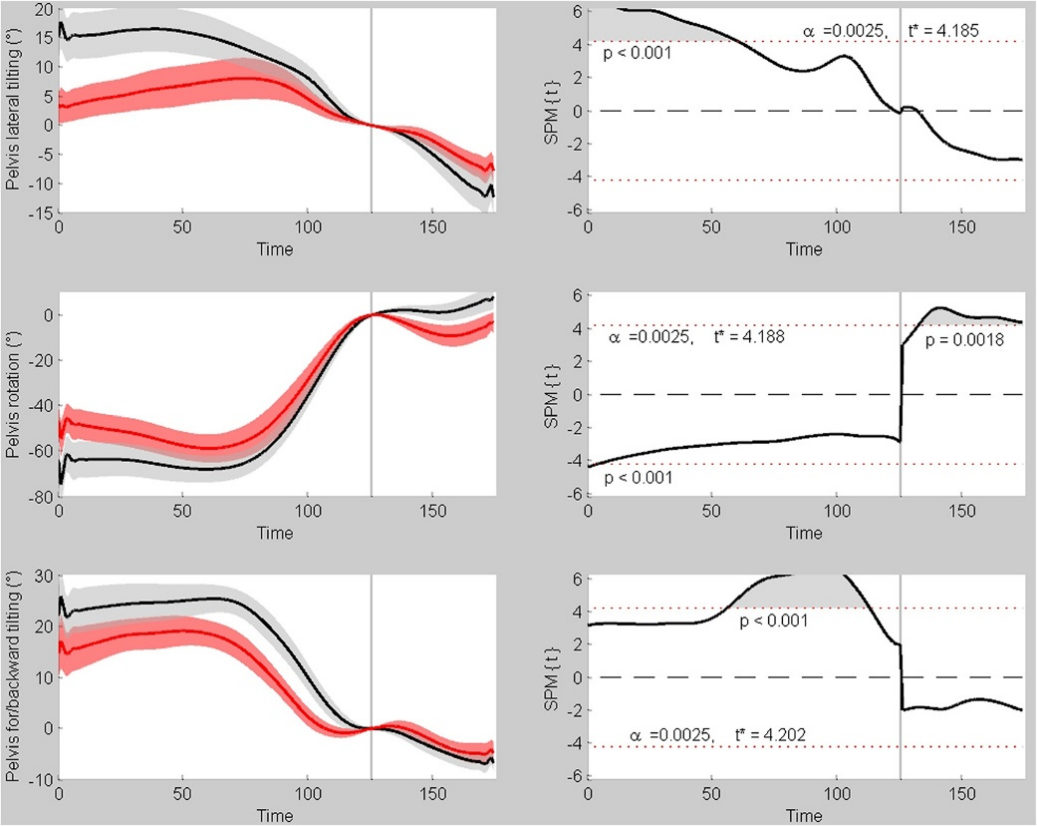
Pelvis kinematics (angles). Mean ± SD time series with their respective SPM{t} fields beside them for pelvis left(−)/right(+) tilting (upper figure), pelvis outward(−)/inward(+) rotation (middle figure) and pelvis forward(−)/backward(+) tilting (lower figure). Black = male, red = female

**Fig. 6:**
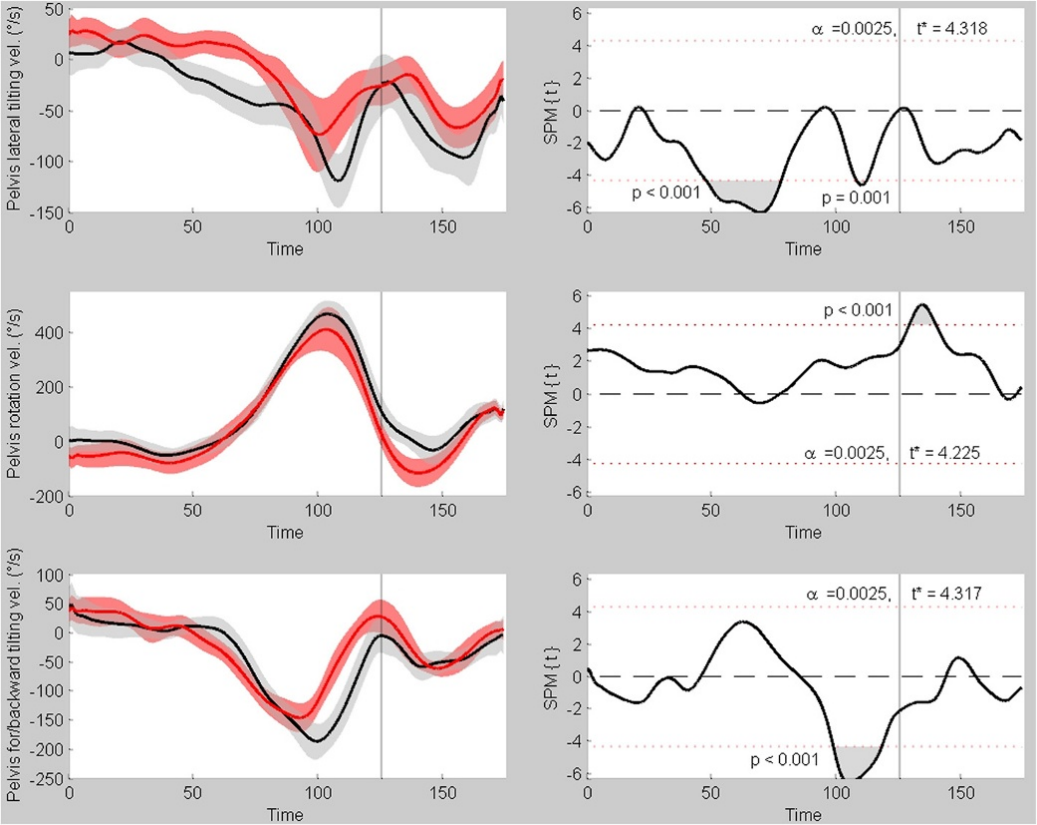
Pelvis kinematics (angular velocities). Mean ± SD time series with their respective SPM{t} fields beside them for pelvis left(−)/right(+) tilting velocity (upper figure), pelvis outward(−)/inward(+) rotation velocity (middle figure) and pelvis forward(−)/backward(+) tilting velocity (lower figure). Black = male, red = female

For the 20 variables that were analyzed with a two-sample SPM{t} test, 12 were found to have significant differences between male and female team handball players. Of these 12 variables, the Mean ± SD time series and their respective SPM{t} fields are shown in Figs. 3, 4, 5, and 6. The vertical lines at time = 125 indicate the point of ball release.

Figure 3 shows the two significant variables from the trunk rotation/velocity profiles. Trunk endo/exorotation velocity showed a significant supra-threshold cluster (*p* < 0.001) from 292 ms till 196 ms prior to ball release. During this time span, the male players clearly showed a higher exorotation velocity than the female players whose time series remained around zero °/s at this time (with a slight tendency towards endorotation velocity). Trunk flexion/extension velocity showed a significant supra-threshold cluster (*p* < 0.001) from 92 ms until 4 ms prior to ball release. The female players exhibited a higher trunk flexion velocity and the corresponding graph also shows a shift to the left indicating an earlier onset timing and an earlier maximal flexion velocity timing than the graph for the male players.

Figure 4 shows the significant variables from the elbow and shoulder rotation/velocity profiles. The angular velocity profile from the elbow revealed two significant supra-threshold clusters (both *p* < 0.001). The first one occurred from 500 ms till 432 ms prior to ball release during which the male players showed a higher extension velocity (this probably started earlier, but this was the cut-off point of our time series). The second cluster was situated from 300 ms till 272 ms prior to ball release. In this time span, the male graph shows an elbow flexion velocity profile while the female graph shows an extension profile (small effect). The shoulder horizontal ab/adduction graph shows two very small, but significant clusters. The first cluster in between 216 ms and 212 ms prior to ball release (*p* = 0.002), the second one is between 180 ms and 176 ms prior to ball release (*p* = 0.003). For both clusters, the male players had higher shoulder horizontal abduction angles. The shoulder internal/external rotation profile showed one significant supra-threshold cluster (*p* < 0.001) that started at 284 ms prior to ball release and ended at 52 ms prior to ball release. During this cluster, the male players showed a much smaller exorotation angle than the female players. The last graph in Fig. 4 (shoulder internal/external rotation velocity) indicates a significant difference (*p* = 0.002) at a single moment in time (140 ms prior to ball release). The male players showed a higher exorotation velocity for this time frame. Figure 5 shows the three angles describing the pelvis orientation in the global reference frame. Pelvis lateral tilting showed a significant gender difference (*p* < 0.001) from 500 ms till 256 ms prior to ball release. The male motion pattern clearly presented a higher lateral tilting to the right than the female pattern. Pelvis rotation showed two supra-threshold clusters. During the first cluster (500 ms till 484 ms prior to ball release, *p* < 0.001), the male players showed a higher outward rotation of the pelvis and during the second cluster (36 ms till 200 ms after ball release, *p* = 0.0018), the male players showed a higher inward rotation. The final graph in Fig. 5 shows the forward/backward tilting of the pelvis. This cluster (*p* < 0.001) started at 228 ms prior to ball release and ended at 48 ms prior to ball release. The male players had a higher backward pelvis rotation during this time span.

Figure 6 shows the pelvis angular velocity variables. Pelvis lateral tilting velocity presented two significant supra-threshold clusters (*p* < 0.001 and *p* = 0.001 respectively). The first one occurred between 304 ms and 188 ms prior to ball release, during which the male players showed a leftward tilting velocity of the pelvis, while the graph of the female players stays around 0°/s at this time span. The second cluster (between 64 ms and 52 ms prior to ball release) is located at the time where the male players reached their maximal leftward tilting velocity, which is higher than the maximal female velocity and lies clearly closer to ball release. Pelvis rotation velocity has one significant cluster between 20 ms and 60 ms after ball release (*p* < 0.001). The graph of the female players shows a higher outward rotation velocity around this cluster period. The final graph in Fig. 6 shows the angular velocity of forward/backward pelvis tilting velocity. A significant supra-threshold cluster of this variable was found between 100 ms and 28 ms prior to ball release (*p* < 0.001). It shows that the male players had a higher forward pelvis tilting velocity during this time interval and that their peak velocity was located closer to ball release than the females.

**Fig. 7:**
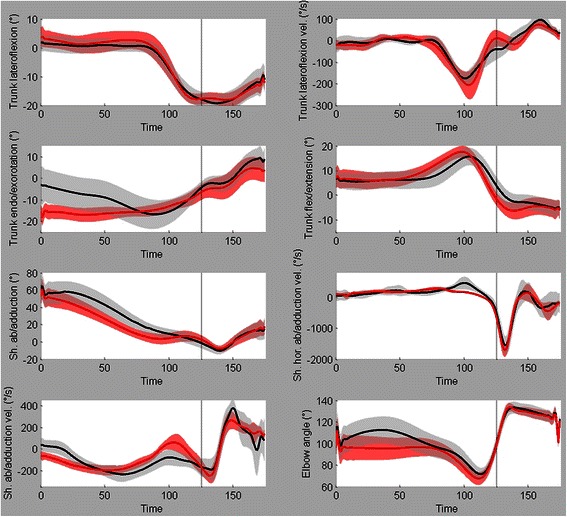
Other kinematic variables. Mean ± SD time series for trunk left(−)/right(+) tilting, trunk left(−)/right(+) tilting velocity, trunk endo(+)/exorotation (−), trunk flexion(−)/extension(+), shoulder ab(+)/adduction(−), shoulder horizontal ab(+)/adduction(−) velocity, shoulder ab(−)/adduction(+) velocity and elbow flexion/extension(full extension = 180°)

The variables that did not reach statistical significance are shown in Fig. 7, but without their respective SPM{t} fields to save space.

## Discussion

The objective of this study was to compare ball release speed and several kinematic parameters between male and female team-handball players. As was hypothesized, male team-handball players showed higher ball release speeds than their female counter-parts and this was not a coincidence of trial-to-trial variation. Based on the results of van den Tillaar & Ettema [3], this difference could be explained by our inability to match both groups on weight and height. Our second hypothesis concerning differences in throwing kinematics between genders was also confirmed by an SPM-analysis (12 out of 20 variables reached significance). These effects were not due to trial-to-trial variation as was confirmed by the mixed model SPM-ANOVA. This can be an additional explanation for the difference in ball release speed. Keeping age, training experience, weight and height matched at the same time for both groups was not possible in this study, so the differences seen in the throwing kinematics could be caused by the differences in body seize. It is possible that the different throwing patterns are the result of specific optimization strategies under the constraints of anthropometric features. Nevertheless, the observed differences can suggest important kinematic features to explain the throwing mechanics and can later be used to design intervention studies. This is not to say that the mean male throwing pattern was better than the female throwing pattern and that athletes should try to imitate a ‘role model pattern’. If these throwing kinematics are in fact influenced by the anthropometric profile, gender-specific guidelines for coordination- and strength training might be in order.

The trunk endo/exorotation velocity time series revealed an interesting gender difference. The higher exorotation velocity in the preparation phase, which was absent for female players, could be a strategy to apply a pre-stretch on the oblique abdominal muscles leading to a more explosive trunk endorotation. Trunk endorotation velocity was indeed higher for male players, but did not reach statistical significance. This result can be compared with the results of Wagner et al. [18] who found significant and positive correlations between ball release speeds and maximal trunk endorotation velocity (r = 0.78) and maximal trunk exorotation angle (r = 0.65). A second gender difference for trunk kinematics was found in trunk flexion/extension velocity during the acceleration phase. The female players exhibited a higher trunk flexion velocity and their timing (onset and maximal velocity) was earlier. Male players reached their peak trunk flexion velocity nearly at ball release, whereas the female players were already accelerating towards trunk extension (countermotion movement).

Elbow angular velocity showed two significant clusters in the preparation phase. Many studies illustrated the importance of maximal elbow extension velocity and elbow extension velocity at ball release [18, 23], but in this study, we were unable to find differences in elbow extension velocity in the acceleration phase. Another variable that is frequently reported in literature to be associated with higher ball release speeds is maximal shoulder endorotation velocity (or velocity at ball release) [18, 23, 26]. We found a difference between the two shoulder rotation velocity curves, but not during the maximal values or around ball release. Figure 4 shows that right before the onset of shoulder endorotation velocity, the male players had a short-term and small-in-magnitude exorotation velocity. This whip-like motion in the throwing shoulder did not lead to higher shoulder endorotation velocities however and thus could not have contributed to ball release speed.

Male players had higher shoulder horizontal abduction angles during the cocking phase, which gives a larger pre-stretch on the Pectoralis Major - and anterior Deltoid muscle fibers. This potential energy created by decelerating the horizontal shoulder abduction motion can be used to accelerate the ball. In shoulder rotation, we see that female players having higher exorotation angles during the cocking phase (pre-stretch on Pectoralis Major and Subscapularis muscles), indicating the difference in the arm cocking maneuver.

All angles and angular velocities describing the pelvis motion reached a significant gender difference at a given point during the time series. Inter-individual differences in approach angle were spatially normalized by expressing all pelvis angles in function of its orientation at ball release (=0°). Pelvis lateral tilting to the right (countermotion tilt) was higher for male players during the preparation phase. Right thereafter, a significant cluster of pelvis leftward tilting velocity (higher for male players) was present. Also around the peak pelvis left tilt velocity, a significant cluster indicated higher values for male players and this peak was located much closer to ball release than for female players. Pelvis rotation showed a significant gender difference after ball release speed, indicating that female players, after ball release, did not exhibit a follow through pelvis rotation. Their pelvis started to rotate backward in exorotation. This is probably because of the lower pelvis rotation velocity during the acceleration phase (although this was not significant). Van den Tillaar & Ettema [26] found a correlation (r = 0.84) between timing of maximal pelvis rotation angle (countermotion angle) and ball release speed, this was confirmed by Wagner et al. [18], r = 0.64. We could not find a difference at this location in time with our sample. Wagner et al. [18] also found a high correlation (r = 0.72) between ball release speed and maximal pelvis internal rotation velocity. Male players had higher pelvis backward tilting angles and higher pelvis forward tilting velocities than female players. The onset of forward pelvis tilting appears to occur around the same time, but the maximal forward pelvis tilting velocity for males occurs closer to ball release. The fact that all pelvis kinematics were significantly different, suggests, that this is a very important segment within the chain of motion. The pelvis serves as a connection between the motion of the legs and the motion of the trunk. A stable, but fast rotating pelvis is necessary for the trunk, the pelvis is used as a base whereon the trunk can start its rotation. Saeterbakken et al. [27] examined the effect of core-stability training on throwing velocity in female team-handball players and found an increased velocity after 6 weeks. They proposed that a stronger and more stable lumbopelvic-hip complex may contribute to higher rotational velocity in multi-segmental movements. However, they did not measure the kinematics of the pelvis (and trunk) motions. This would be a very interesting topic and can give more insights into the actual role of the pelvis.

**Fig. 8:**
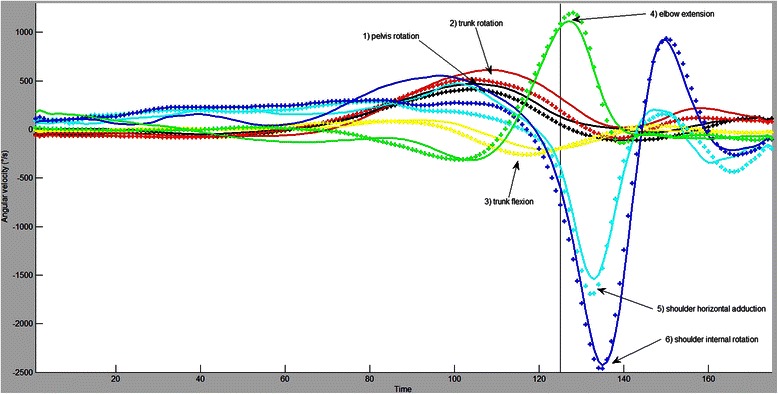
Proximal-to-distal sequencing in male (−−−) and female (+++) team-handball throwing in terms of angular velocity profiles. The sequence of the maximal angular velocities follows the typical team-handball proximal-to-distal sequence of pelvis rotation, trunk rotation, trunk flexion, elbow extension, shoulder horizontal adduction and shoulder internal rotation

From the previous discussion, it is clear that male and females throwers differ in generating momentum and transferring it from the ground through the kinematic chain towards the throwing arm and finally to the ball (we did not study the kinematics of the final joint, the wrist). Male team-handball players showed more activity in the transverse plane (pelvis and trunk rotation and shoulder horizontal abduction) whereas female team-handball players showed more activity in the sagittal plane (trunk flexion). An important aspect in the transfer of momentum is proximal-to-distal sequencing [8–10]. An analysis with SPM offers many advantages (no data reduction and thus no directed hypothesis testing and an easier graphical way to communicate results of 1D time series), but a clear distinction within a cluster, of differences in magnitudes or timing would need post-hoc scalar tests. The team-handball specific proximal-to-distal sequence as indicated by maximal joint velocities (starting with pelvis rotation, followed by trunk rotation, trunk flexion, elbow extension, shoulder horizontal adduction and shoulder internal rotation) was also observed in our data as illustrated in Fig. 8. Most gender differences were observed in the early and late preparation phases of the throw. This period is probably the most important, because in this phase, the build-up of momentum from the pelvis and trunk (with their large masses) is maximal. At the end of the preparation phase, the reversal of the torques working on the pelvis and trunk will create a torque on the shoulder [28] and the transfer of momentum to the throwing arm will occur. Even more gender differences might be apparent in the standing throw with run-up, but did we not detect them. We have set our significance threshold at a fairly conservative level (Bonferroni correction; indicating 20 independent variables, which is of course not the case). This needs to be taken into account when interpreting the results, because within a kinematic chain, all variables have a time-varying covariance and are thus not independent. Further research needs to be done to determine these interrelations between variables.

The standing throw with run-up that was analyzed in this study is the second most popular throwing technique (14–18 %) in team-handball [29], but it is the throwing technique which produces the highest ball release speed [18]. In future studies, this gender difference should be confirmed in the jump shot which is the most popular throwing technique in team-handball games [29]. An additional limitation of this study is the low sample size, only 10 players from each gender participated. In future studies, this number should be higher to extrapolate to a larger population. This study was situated within a deterministic framework [11] where we studied differences in kinematic time series and ball release speed. Future studies are needed to assess see if these differences are caused by the anthropometric profile and if they can be used to increase throwing speed. The relationships among all variables are not taken into account and are not studied in one statistical test. Multivariate techniques in SPM can be a solution to this problem and will be a very interesting research area in the future. Also other techniques in the field of mechanics such as Induced Acceleration Analysis [30] and in the field of pattern recognition such as Neural Networks [31, 32] could give further insights into throwing mechanics and coordination in team-handball and other overhead throwing sports.

## Conclusions

We can conclude that, in contrast to previous research [4], gender differences in throwing kinematics are present. The exact nature of the role of kinematics in the relation between anthropometric profile and ball speed still remains unclear. Differences were found in the orientation and velocities of pelvis, trunk, shoulder and elbow kinematic time series. We could say that male players showed more activity in the transverse plane while female players showed more activity in the sagital plane. Based on our results, the motion of the pelvis may be serving as a more important segment than was previously stated. As stated by Wagner et al. [12], coaches can easily observe the motions of pelvis and trunk because these are the largest segments and rotate at a relatively lower angular velocity. Coordination training (especially in youth training) should focus more on pelvis and trunk motions. Also, future research should examine the effect of different types of training such as core-stability/core-strength training and differential training on pelvis- and trunk kinematics to establish a causal link between differences in throwing pattern and differences in ball release speed.

Statistical Parametric Mapping clearly offers an advantage for sports biomechanics, because it allows us to use the entire data-sets and thus, more differences may be observed that would slip past researchers if they would extract only minima/maxima.
